# GreenCircRNA: a database for plant circRNAs that act as miRNA decoys

**DOI:** 10.1093/database/baaa039

**Published:** 2020-06-08

**Authors:** Jingjing Zhang, Zhiqiang Hao, Shuwei Yin, Guanglin Li

**Affiliations:** 1 Key Laboratory of Ministry of Education for Medicinal Plant Resource and Natural Pharmaceutical Chemistry, Shaanxi Normal University, West Chang'an Street, Xi'an 710062, China; 2 College of Life Sciences, Shaanxi Normal University, West Chang'an Street, Xi'an 710062, China

## Abstract

Circular RNAs (circRNAs) are endogenous non-coding RNAs that form a covalently closed continuous loop, are widely distributed and play important roles in a series of developmental processes. In plants, an increasing number of studies have found that circRNAs can regulate plant metabolism and are involved in plant responses to biotic or abiotic stress. Acting as miRNA decoys is a critical way for circRNAs to perform their functions. Therefore, we developed GreenCircRNA—a database for plant circRNAs acting as miRNA decoys that is dedicated to providing a plant-based platform for detailed exploration of plant circRNAs and their potential decoy functions. This database includes over 210 000 circRNAs from 69 species of plants; the main data sources of circRNAs in this database are NCBI, EMBL-EBI and Phytozome. To investigate the function of circRNAs as competitive endogenous RNAs, the possibility of circRNAs from 38 plants to act as miRNA decoys was predicted. Moreover, we provide basic information for the circRNAs in the database, including their locations, host genes and relative expression levels, as well as full-length sequences, host gene GO (Gene Ontology) numbers and circRNA visualization. GreenCircRNA is the first database for the prediction of circRNAs that act as miRNA decoys and contains the largest number of plant species.

**Database URL**: http://greencirc.cn

## Introduction

Circular RNAs (circRNAs) have been a hot topic in non-coding RNA research in recent years. First discovered in the 1970s (1, 2), circRNAs are characterized by covalently closed-loop structures with neither a 5′ cap nor a 3′ polyadenylated tail and are generated by back splicing (3). Because of their special cyclic structure, circRNAs are insusceptible to degradation by RNA exonuclease or RNase R, which suggests that these RNAs have important functions *in vivo* (4, 5). With the development of high-throughput RNA sequencing, thousands of circRNAs have been identified in humans, mammals and fungi (6–8). Moreover, many circRNAs have been identified in plants, including *Arabidopsis thaliana* (9), maize (10), tomato (11), wheat (12) and rice (13). Recent studies found that plant circRNAs play regulatory roles in stress response (14). For instance, Vv-circATS1 responds to cold stress in grape (15), and overexpression of circR5g05160 can improve the resistance of rice to *Magnaporthe oryzae* (16). MicroRNAs (miRNAs) are a class of non-coding RNAs that are approximately 20–24 nucleotides in length (17) and interact with mRNAs to regulate gene expression (18). Studies have shown that circRNAs are able to sequester and inactivate miRNAs by acting as miRNA decoys (also known as miRNA sponges) (19–21), for which function circRNAs carry a short stretch of sequences that share homology with miRNA binding sites in endogenous mRNA targets. For example, circRNA ciRS-7 has 73 conserved miR-7-binding sites and strongly suppresses miR-7 activity by acting as a miR-7 decoy, affecting expression levels of miR-7 targets (22–24). Currently, many studies are also focusing on the function of circRNA as a miRNA decoy in plants. For instance, 102 circRNAs were found to act as decoys for 24 corresponding miRNAs in tomato (11), 6 circRNAs that respond to dehydration were found to act as decoys for 26 corresponding miRNAs in wheat (12) and 346 circRNAs were found to act as decoys in *Zea mays* (25). Thus, inferring the function of plant circRNAs based on circRNAs as miRNA decoys is an effective approach.

**Figure 1:**
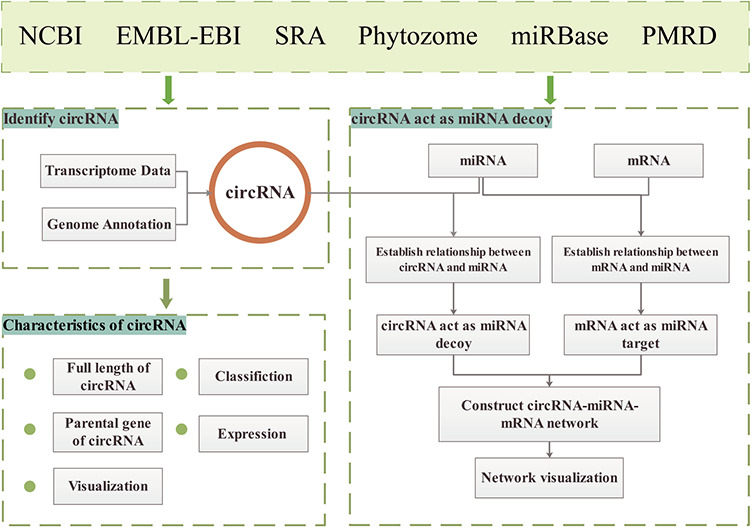
GreenCircRNA framework.

With the huge progress in animal circRNAs research, some animal circRNA databases have been established, such as CircNet, Circ2Traits and CircRNADb (26–28). In contrast, with the advance of plant circRNAs, there are only two databases of plant circRNAs that have been built, namely, PlantCircNet and PlantcircBase (29, 30), but there is no database for identifying circRNAs as miRNA decoys in plants. In this study, we established Green CircRNA—a database for plant circRNAs that act as miRNA decoys. This database contains 69 plants in total, with 38 plants having relevant miRNA information. Based on the information available, we investigated circRNAs as miRNA decoys and identified mRNAs as miRNA targets in these 38 plants. These findings will facilitate further analysis of the function of circRNAs as competitive endogenous RNAs. GreenCircRNA is a comprehensive plant circRNA database containing 213 494 circRNAs from 69 plant species using 4116 transcriptome sequencing data sets and provides relevant information, full-length sequences and regulatory networks for those circRNAs. We believe that GreenCircRNA is a comprehensive and valuable resource and an important platform for further research on plant circRNAs. The data set used by our database can be downloaded freely. Henceforth, we will continue to supplement the data in GreenCircRNA (http://greencirc.cn).

### Aims of the database

CircRNAs play essential roles in regulating plant development and metabolism. The mechanism of formation, function and conservation of circRNAs in plants is the focus of recent circRNA research (6, 8, 31). To gain a deeper understanding of the mechanism and functions of circRNAs as miRNA decoys in plants, we constructed a comprehensive database for plant circRNAs that act as miRNA decoys. This database was created with three main goals: (i) use of publicly available high-throughput transcriptome sequencing data to identify circRNAs in various plants and archive related information for these circRNAs, (ii) analysis of the potential function of circRNAs by identifying circRNAs that act as miRNA decoys and (iii) provision of a user-friendly website with useful web-based tools for the investigation of plant circRNAs.

**Figure 2:**
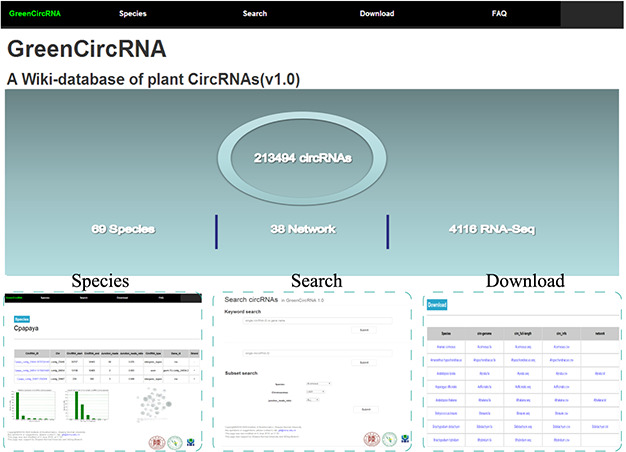
Web interface.

### Materials and Methods Data collection and implementation

The GreenCircRNA database website is built on a `LAMP' (Linux, Apache, MySQL and PHP) open-source architecture. The principal data sources of GreenCircRNA are NCBI (https://www.ncbi.nlm.nih.gov/) (32), EMBL-EBI (https://www.ebi.ac.uk/), SRA database, Phytozome (https://phytozome.jgi.doe.gov/pz/portal.html), miRBase (33) (www.mirbase.org/) and Plant MicroRNA Database (PMRD: http://bioinformatics.cau.edu.cn/PMRD/) (34) (Figure 1). We downloaded plant genome data and annotation information from Phytozome12; transcriptome sequence data, miRNA sequences and mRNA sequences were collected from NCBI, EMBL-EBI, miRBase andPMRD.

#### Identification of circRNAs and related information

After obtaining all types of data, we identified circRNAs from various plants using CIRI (v2.0.6) and CIRCexplorer2 (v2.3.3) (35–38). In addition, we provide related information regarding circRNAs in the database, including the following:

(1) Location of the circRNA in the genome;
(2) Junction read number of circRNAs and junction read IDs;
(3) Relative expression of the circRNA calculated by [2*junction_reads/(2*junction_reads + non-junction_reads)];
(4) Classification of circRNAs (exon, intron and intergenic region);
(5) Host gene of circRNAs and GO (Gene Ontology) number of the host gene (excluding intergenic region circRNAs);
(6) SRA accession number that generated the circRNAs;
(7) Sequence of the circRNA extracted from the start position to the end position (named circ-genome-seq).

**Table 1:** Statistics of GreenCircRNA database

Species	Number of circRNAs	Number of exon circRNAs	Number of intergenic region circRNAs	Number of intron circRNAs	Number of circRNAs full-length
*Amaranthus hypochondriacus*	1536	547	872	117	0
*Ananas comosus*	723	220	428	75	178
*Arabidopsis lyrata*	2886	623	1032	1231	0
*Arabidopsis thaliana*	10 707	5254	554	4899	2469
*Asparagus officinalis*	1303	379	767	157	962
*Botryococcus braunii*	372	90	236	46	123
*Brachypodium distachyon*	555	229	294	32	227
*Brachypodium hybridum*	288	94	190	4	104
*Brachypodium stacei*	75	22	49	4	24
*Brachypodium sylvaticum*	2590	617	526	1447	541
*Brassica oleracea capitata*	2113	415	678	1020	399
*Brassica rapa FPsc*	124	10	54	60	5
*Capsella grandiflora*	2517	1885	597	35	1357
*Capsella rubella*	4121	2575	546	1000	1605
*Carica papaya*	3984	1200	801	1983	1089
*Chenopodium quinoa*	3755	410	3183	162	611
*Chlamydomonas reinhardtii*	2825	1142	258	1425	93
*Chromochloris zofingiensis*	253	89	155	9	7
*Cicer arietinum*	1345	796	511	38	253
*Citrus clementina*	1016	162	440	414	145
*Citrus sinensis*	1829	208	355	1266	176
*Cucumis sativus*	5432	3992	1169	271	1472
*Daucus carota*	1053	497	518	38	327
*Dunaliella salina*	42	19	16	7	9
*Eucalyptus grandis*	3253	287	2901	65	284
*Eutrema salsugineum*	248	103	145	0	119
*Fragaria vesca*	6352	1341	1025	3986	1080
*Glycine max*	7938	2952	925	4061	1850
*Gossypium hirsutum*	3988	2598	1291	99	846
*Gossypium raimondii*	652	189	269	194	195
*Helianthus annuus*	11 858	6628	4753	477	5141
*Hordeum vulgare*	1162	504	341	317	163
*Kalanchoe fedtschenkoi*	1106	159	907	40	35
*Lactuca sativa*	5540	3996	1429	115	2936
*Linum usitatissimum*	2022	462	495	1065	0
*Malus domestica*	3141	653	565	1923	219
*Manihot esculenta*	4543	2559	1906	78	2190
*Marchantia polymorpha*	707	498	204	5	479
*Medicago truncatula*	5668	2244	1670	1754	1580
*Micromonas pusilla CCMP1545*	6119	3102	2936	81	5180
*Micromonas sp.RCC299*	151	87	58	6	48
*Mimulus guttatus*	1296	747	535	14	664
*Musa acuminata*	2504	1017	1400	87	995
*Olea europaea*	860	50	786	24	77
*Oryza sativa*	17 479	6684	1405	9390	3931
*Oryza sativa Kitaake*	1050	509	478	63	340
*Panicum hallii*	1578	561	961	56	254
*Panicum virgatum*	3296	441	2743	112	408
*Phaseolus vulgaris*	8866	6241	2503	122	5856
*Physcomitrella patens*	430	267	110	53	283
*Populus deltoides WV94*	661	122	527	12	127
*Populus trichocarpa*	2090	229	1834	27	248
*Porphyra umbilicalis*	663	447	210	6	378
*Prunus persica*	2925	1976	916	33	1420
*Ricinus communis*	2861	859	1264	738	976
*Salix purpurea*	2310	589	1637	84	301
*Setaria italica*	2099	1388	665	46	909
*Setaria viridis*	1514	1033	440	41	833
*Solanum lycopersicum*	2426	1225	1090	111	1485
*Solanum tuberosum*	6644	1136	3447	2061	880
*Sorghum bicolor*	1165	540	586	39	436
*Spirodela polyrhiza*	83	15	66	2	38
*Theobroma cacao*	4443	3169	696	578	664
*Trifolium pratense*	1792	759	919	114	390
*Triticum aestivum*	3182	2249	679	254	2330
*Vigna unguiculata*	5767	4636	1097	34	3778
*Vitis vinifera*	7412	5067	951	1394	2974
*Zea mays*	12 035	3111	1118	7806	2630
*Zostera marina*	171	106	63	2	111

#### CircRNAs as miRNA decoys

Functional research on circRNAs is the main challenge in the study of plant circRNAs. To infer the potential functions of circRNAs, we established a circRNA–miRNA–mRNA network in which circRNAs act as miRNA decoys and mRNAs act as miRNA targets. First, we downloaded miRNA sequences from miRBase and PMRD and mRNA sequences from Phytozome12 and extracted circRNA sequences by using an in-house Perl script. Next, to establish the relationship between miRNAs and other RNAs, we used RNAplex with default parameters to predict RNA–RNA hybridization sites (39, 40). We then identified miRNA decoys (circRNAs) and targets (mRNA) following a method proposed in our previous report (41–43, 25). The criteria used to define a miRNA decoy were as follows: no more than six mismatched or inserted bases present between the 9th and 20th nucleotides of the miRNA 5′ end, perfect matching of the second to eighth bases of the miRNA 5′ end sequence and no more than four mismatches or indels in other regions. The criteria used to define a miRNA target were as follows: at most, one mismatch or indel was allowed between the 9th and 12th positions of the 5′ end of miRNA sequences, the total number of bulges or mismatches in the other regions was not allowed to exceed 4 nt and no continuous mismatches were allowed. Finally, a picture of the circRNA–miRNA–mRNA network for a species was generated using Cytoscape (v3.7.2) (44). The influence of circRNAs on other mRNAs via miRNAs can be assessed according to the circRNA–miRNA–mRNA network, and the potential functions of the circRNAs can be inferred.

#### Full-length sequences of circRNAs

The full-length sequences of circRNAs are important for subsequent analysis of the internal structural features and functions of circRNAs, which can help in evaluating the translation potential of circRNAs. However, most circRNA databases do not provide the full-length sequences of circRNAs. For our database, we used downstream programs of CIRI, named CIRI-full, to assemble the full-length sequences of circRNAs (45). After removing redundant results with the in-house Perl script, we obtained final circRNA full-length sequences.

#### CircRNA visualization

CircRNAs form covalently closed-loop structures with neither 5′-3′ polarities nor polyadenylated tails. As providing the position of circRNAs in the genome does not lead to an intuitive understanding of its structure for users, a schematic of each circRNA is available in the database. The schematic consists of two parts: one is a line of the host gene of the circRNA, with the exons and introns in the gene labeled; the other is a circle of the circular structure of circRNA, which is marked with corresponding colors that are the same as the position on the gene. Thus, the position and structure of the circRNA can be observed visually, which makes the information easy to understand.

**Figure 3:**
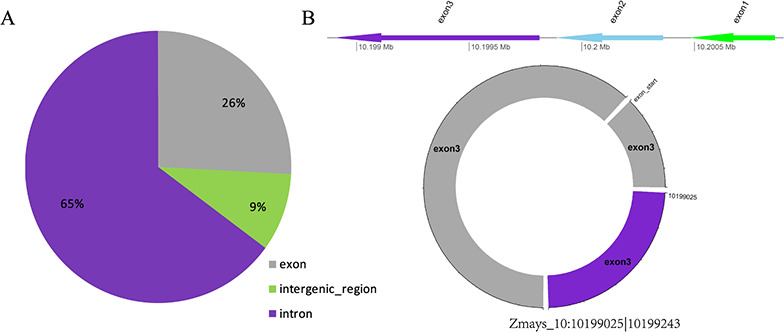
(A) Distribution of three types of circRNAs in maize. (B) Visualization of the circRNA Zmays_10:10199025|10 199 243.

**Figure 4:**
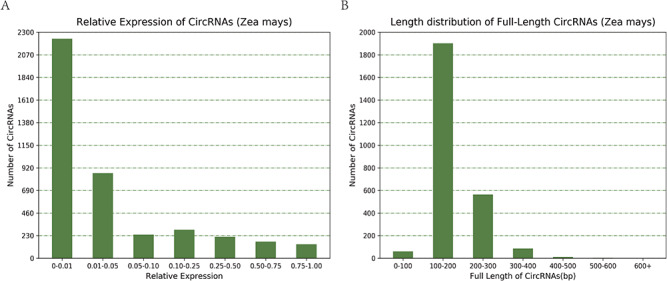
(A) Relative expression of circRNAs in Zea mays. (B) Length distribution of full-length circRNAs in Zea mays.

**Figure 5:**
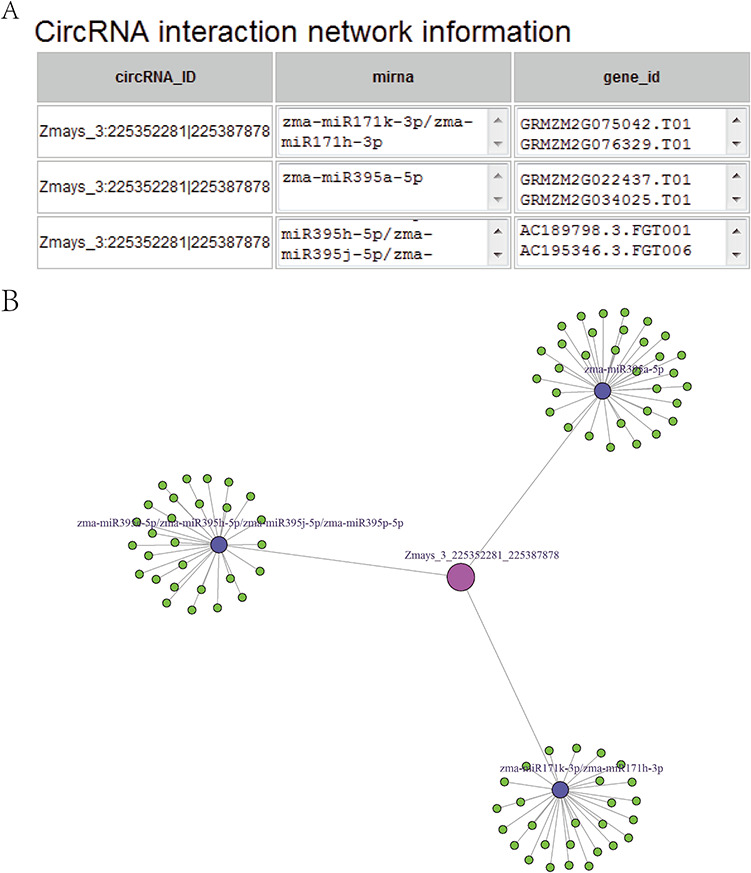
(A) CircRNA–miRNA–mRNA sub-network, taking circRNA Zmays_3:225352281|225 387 878 as an example. Blue nodes, miRNA. Pink node, circRNA as a miRNA decoy. Green nodes, mRNAs as miRNA targets. Gray edges, correlations. (B) Sequence list for Figure 5A, taking circRNA Zmays_3: 225352281|225 387 878 as an example. The first column is a circRNA that acts as a miRNA decoy, the second column shows miRNAs and the third column contains mRNAs that act as miRNA targets. This circRNA may act as a decoy for three miRNAs: zma-miR171k-3p/zma-miR171h-3p, zma-miR395a-5p and zma-miR395e-5p/zma-miR395h-5p/zma-miR395j-5p/zma-miR395p-5p.

## Results

### Usage and access

GreenCircRNA mainly includes the following modules: Home, Species, Search, Download and FAQ (Figure 2). Users can browse, search and download circRNA information through the web interface of GreenCircRNA.

#### Species

Basic information of many circRNAs belonging to 69 plant species is included in this module. There is an individual interface of each plant containing detailed circRNA associated information, including a list of all circRNAs in this plant, a histogram showing the relative expression levels of all circRNAs, a length distribution histogram of the circRNA full-length sequences and a circRNA–miRNA–mRNA network illustrating the potential relationships of these RNAs. Furthermore, there is a separate page for every circRNA that displays detailed information for the circRNA, including the location of the circRNA in the genome, the relative expression level, the full-length sequence, the host gene and annotation information. Users can obtain all information for a given circRNA on this page, and this page also provides a circRNA network that shows circRNAs as miRNA decoys in a tabular and graphical manner.

#### Search

The module enables users to search for circRNAs by host gene, miRNA ID, circRNA ID and SRA ID. In addition, a subset search is available in this module, and users can search circRNAs by a series of criteria, such as plant species name, chromosome and relative expression level.

#### Download

Related information for circRNAs, including basic information, circ-genome-seq, full-length sequences and network, can all be downloaded for free in CSV format or fasta format from the `download’ module.

### Data summary

The GreenCircRNA database covers 69 plant species, which is the highest number of plant species in all plant circRNA databases to date. We downloaded a total of 4116 transcriptome sequencing data sets from SRA and EMBL-EBI for circRNA identification and eventually obtained 213 494 circRNAs. These circRNAs were classified into three categories: exon circRNAs, intron circRNAs and intergenic region circRNAs. Among all the identified circRNAs, 95 010 (44.50%) belong to the exon category, 65 175 (30.53%) belong to the intergenic region category and 53 309 (24.97%) belong to the intron category. We then extracted the sequences of all circRNAs from genome sequences (circ-genome-seq). Furthermore, we assembled 68 237 full-length sequences of circRNAs using CIRI-full and predicted the conditions that circRNAs act as miRNA decoys for 38 plant species (Table 1).

### A case study

Taking maize as an example, we downloaded 181 transcriptome sequencing data sets from the SRA and EMBL-EBI databases for circRNA identification and obtained 12 035 circRNAs in total, including 3111 (25.85%) belonging to exons, 7806 (64.86%) belonging to introns and 1118 (9.29%) belonging to intergenic regions (Figure 3A). By contrast, the results of software CIRI predicted more exon circRNAs, and the result of software CIRCexplorer predicted more intron circRNAs. In addition, 2630 full-length sequences of circRNAs in maize were assembled by CIRI-full; the relative expression levels and length distributions of the circRNA full-length sequences are displayed in histograms (Figure 4A and B). Moreover, an individual page shows detailed information for each circRNA, including the location in the genome, the relative expression level and visualization. For instance, the exon circRNA Zmays_10:10199025|10 199 243 is located in the middle of the third exon of the gene `GRMZM2G046284.v6a’ (Figure 3B). We analyzed circRNAs that may act as miRNA decoys in maize and showed their relationships by the circRNA–miRNA–mRNA network and a table that includes these RNAs involved in the network on the single circRNA page. For example, circRNA Zmays_3:225352281|225 387 878 may act as decoys for three miRNAs: zma-miR171k-3p/zma-miR171h-3p, zma-miR395a-5p and zma-miR395e-5p/zma-miR395h-5p/zma-miR395j-5p/zma-miR395p-5p (Figure 5).

## Discussion and future prospects

Increasing evidence has proven that circRNAs play important roles in various biological processes, but few studies have examined circRNAs in plants. Furthermore, specific circRNA data for analyzing the sequence and structure of circRNAs are not available for most plant species, and the functions and mechanisms of most circRNAs are unclear. Although two databases of plant circRNAs have been built, these databases cover relatively fewer plant species. In this study, we developed GreenCircRNA, a comprehensive database of plant circRNAs that includes circRNAs in 69 plants, and analyzed the potential decoy function of these circRNAs in 38 plants. Users can freely search and download information related to circRNAs. We hope that as a platform, GreenCircRNA will help researchers to study the basic properties and characteristics of plant circRNAs and will be useful for further research on the internal structure, translation function and mechanism of circRNAs. This database can still be improved, and we will continuously identify and collect more circRNAs and update and improve GreenCircRNA to provide accurate information regarding plant circRNAs and circRNAs as miRNA decoys.
